# Lytic cell death induced by melittin bypasses pyroptosis but induces NLRP3 inflammasome activation and IL-1*β* release

**DOI:** 10.1038/cddis.2017.390

**Published:** 2017-08-10

**Authors:** Fátima Martín-Sánchez, Juan José Martínez-García, María Muñoz-García, Miriam Martínez-Villanueva, José A Noguera-Velasco, David Andreu, Luís Rivas, Pablo Pelegrín

**Affiliations:** 1 Inflammation and Experimental Surgery Unit, Biomedical Research Institute of Murcia IMIB-Arrixaca, University Clinical Hospital Virgen de la Arrixaca, Murcia 30120, Spain; 2 Clinical Laboratory, University Clinical Hospital Virgen de la Arrixaca, Murcia 30120, Spain; 3 Department of Experimental and Health Sciences, Pompeu Fabra University, Barcelona Biomedical Research Park, Barcelona 08003, Spain; 4 Department of Physico-Chemical Biology, Centro de Investigaciones Biológicas (C.S.I.C.), Madrid 28040, Spain

## Abstract

The nucleotide-binding domain and leucine-rich repeat-containing receptor with a pyrin domain 3 (NLRP3) inflammasome is a sensor for different types of infections and alterations of homeostatic parameters, including abnormally high levels of the extracellular nucleotide ATP or crystallization of different metabolites. All NLRP3 activators trigger a similar intracellular pathway, where a decrease in intracellular K^+^ concentration and permeabilization of plasma membrane are key steps. Cationic amphipathic antimicrobial peptides and peptide toxins permeabilize the plasma membrane. In fact, some of them have been described to activate the NLRP3 inflammasome. Among them, the bee venom antimicrobial toxin peptide melittin is known to elicit an inflammatory reaction via the NLRP3 inflammasome in response to bee venom. Our study found that melittin induces canonical NLRP3 inflammasome activation by plasma membrane permeabilization and a reduction in the intracellular K^+^ concentration. Following melittin treatment, the apoptosis-associated speck-like protein, an adaptor protein with a caspase recruitment domain (ASC), was necessary to activate caspase-1 and induce IL-1*β* release. However, cell death induced by melittin prevented the formation of large ASC aggregates, amplification of caspase-1 activation, IL-18 release and execution of pyroptosis. Therefore, melittin-induced activation of the NLRP3 inflammasome results in an attenuated inflammasome response that does not result in caspase-1 dependent cell death.

Inflammasomes are multiprotein complexes activated in response to multiple pathogen and noninfectious stimuli, including pore-forming toxins, venoms, changes in extracellular osmolarity or extracellular accumulation of metabolites such as ATP or crystalline uric acid.^1, 2, 3, 4, 5^ Inflammasomes are formed by a sensor protein that usually belongs to the nucleotide-binding domain and leucine-rich repeat-containing receptor (NLR) family, among them, the sensor NLR with a pyrin domain 3 (NLRP3), the inflammasome most thoroughly investigated and the only one able to respond to sterile stimulation.^1, 2^ The canonical NLRP3 inflammasome is formed by the sensor protein NLRP3, the adaptor protein apoptosis-associated speck-like protein with a caspase recruitment domain (ASC) and the effector protease caspase-1.^1, 2, 6^ NLRP3 is activated by a wide plethora of stimuli that induce a common intracellular signaling, including a decrease in intracellular K^+^, production of reactive oxygen species, plasma membrane permeabilization, lysosomal destabilization and metabolic disruption.^6, 7, 8^ NLRP3 inflammasome induces ASC assembly and oligomerization by a prion-like process into large aggregates that amplify caspase-1 activation.^9, 10, 11^ Caspase-1 induces maturation and release of the pro-inflammatory cytokines IL-1*β* and IL-18 through a noncanonical (endoplasmic reticulum- and Golgi-independent) release pathway.^12^ The release of these cytokines is mainly due to plasma membrane permeabilization,^13^ induced by the processing of gasdermin D by caspase-1 and by insertion of the lytic amino-terminal fragment in the plasma membrane.^14, 15, 16^ This process also results in a specific type of cell death termed *pyroptosis*, characterized by the release of intracellular content due to plasma membrane permeabilization.^14, 15, 16^

Antimicrobial peptides form a chemical barrier against invading pathogens. These peptides must be massively inserted into the targeted membrane to attain their lethal mechanism, a process facilitated by their amphipathic nature. In many cases, the final step is severe membrane distortion or pore formation in the target cell, with lethal leakage of intracellular metabolites and ions.^17^

To avoid indiscriminate cellular lysis, the targeted microorganism must be recognized. Most antimicrobial peptides are strongly cationic and, therefore, preferentially accumulate in bacterial and lower eukaryote plasma membranes with exposure of anionic phospholipids to the external medium. In higher eukaryotes, acidic phospholipids are confined on the cytoplasmic side of the membrane, exposing a zwitterionic surface to the external medium. Accordingly, antimicrobial peptides specificity relies on a subtle structural blend of amphipaticity and cationic character. In fact, these characteristics are shared with other eukaryotic peptide toxins, such as melittin, endowed with antimicrobial activity aside from their toxic effect on higher eukaryotic cells.^18^ Similarly, at higher concentrations antimicrobial peptides can also permeabilize the plasma membrane of mammalian cells, and it is known that the human antimicrobial peptide LL-37 (a 37-amino acid peptide) and the insect peptide toxin melittin (a 26-amino acid cationic peptide) may be able to release IL-1*β*.^5, 19^ It is also known that LL-37 modulates NLRP3 inflammasome by amplification of purinergic P2X7 receptor response to extracellular ATP;^19, 20^ however, little is known about the mechanism of NLRP3 activation induced by melittin. Melittin is the main component of bee venom, and venoms cause an allergic reaction characterized by local inflammation consisting of redness, pain, heat and swelling.^21^ The NLRP3 inflammasome and IL-1*β* are important for this inflammatory response *in vivo.*^5^ Melittin has a hydrophobic amino-terminal region (1–20 amino acids) and a hydrophilic cationic carboxyl-terminal region (21–26 amino acids); this amphipathic nature allow melittin to be a potent hemolytic molecule by interacting and permeabilizing eukaryotic membranes forming pores.^22, 23^ In this study, we aimed to investigate the mechanism of melittin-induced NLRP3 inflammasome activation. We found that melittin induces K^+^ efflux from macrophages and NLRP3 inflammasome formation. This NLRP3 inflammasome requires the adaptor protein ASC to activate caspase-1 and induce IL-1*β* release; however, melittin was unable to induce large ASC aggregates. Melittin-induced plasma membrane permeabilization and subsequent cell death was independent of caspase-1, showing that rapid cell lysis driven by melittin excludes pyroptosis execution by caspase-1.

## Results

### Melittin, but no other antimicrobial peptides, induces robust release of IL-1*β*

**Figure 1:**
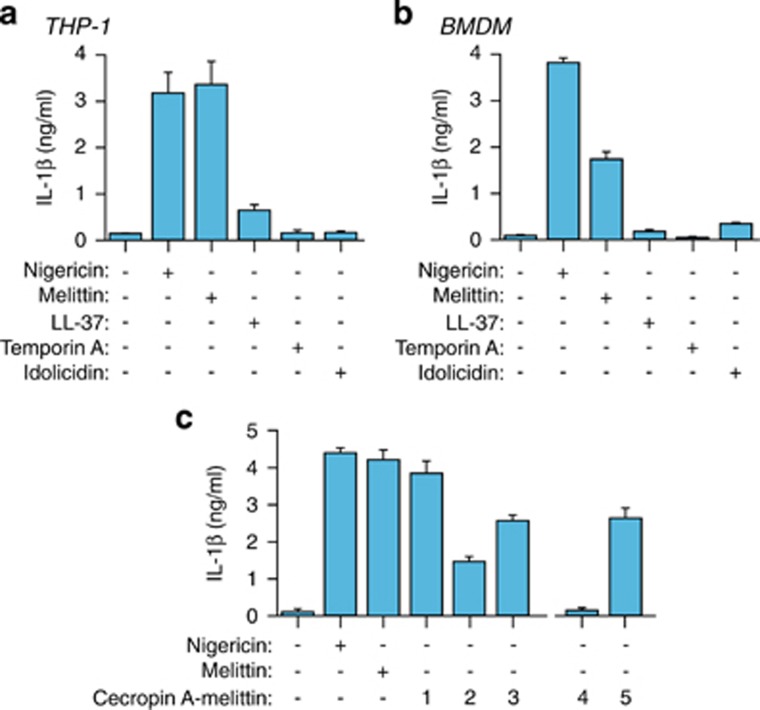
Melittin induces the release of IL-1*β*. (**a** and **b**) ELISA for IL-1*β* release from THP-1 (**a**) or BMDMs (**b**) primed with LPS (1 *μ*g/ml, 4 h), and then stimulated or not for 30 min with nigericin (25 *μ*M), melittin, LL-37, temporin A or indolicidin (10 *μ*M each). (**c**) ELISA for IL-1*β* release from THP-1 treated as in panel (**a**) but with the chimeric peptides shown (10 *μ*M each); chimeric peptides are described in Table 1

Cationic amphipathic antimicrobial peptides may permeabilize plasma membrane and are candidates for NLRP3 inflammasome activation. Different antimicrobial peptides were tested at 10 *μ*M on macrophages for IL-1*β* release (Table 1), with melittin being the only one able to induce robust release of IL-1*β* in both human THP-1 and mouse BMDM macrophages (Figures 1a and b). In THP-1, melittin-induced IL-1*β* release was similar to treatment with nigericin (Figure 1a), a classical NLRP3 inflammasome activator. In contrast, at this concentration, LL-37 – a human peptide reported to induce IL-1*β* release^19^ – induced only scant IL-1*β* release from THP-1 but none from mouse BMDMs (Figures 1a and b). Temporin A and indolicidin failed to trigger significant secretion of IL-1*β* from macrophages (Figures 1a and b), although indolicidin did have strong effects on the membrane of mammalian cells.^24^ To gain further insight into the melittin sequence responsible for IL-1*β* release, we tested chimeric peptides comprising different stretches of the hydrophobic melittin sequence fused to the N-terminus of the antimicrobial peptide cecropin A (Table 1).^25^ These chimeric peptides improve the microbicidal activity of cecropin A, which is otherwise unable to kill Gram-positive bacteria, while decreasing melittin toxicity by excluding the hemolytic part of melittin.^25^ All chimeric peptides (peptides 1 to 3) resulted in IL-1*β* release (Figure 1c), suggesting that the smallest melittin motif necessary to trigger IL-1*β* release is VLKVL (residues 5–9) (Table 1). IL-1*β* release was not attained when disturbing the alpha helix conformation of melittin by replacing the native residues in the VLKVL motif by d-amino acids (Figure 1c and Table 1, peptide 4). However, similar IL-1*β* induction to that of all l-amino acid control peptides did occur when d-amino acids were introduced in the cecropin A moiety of chimeric peptide 5 (Figure 1c and Table 1). This suggests that in chimeric peptides the melittin sequence, rather than the cecropin A region, is responsible for inducing IL-1*β* release.

### Bee venom induces IL-1*β* release

**Figure 2:**
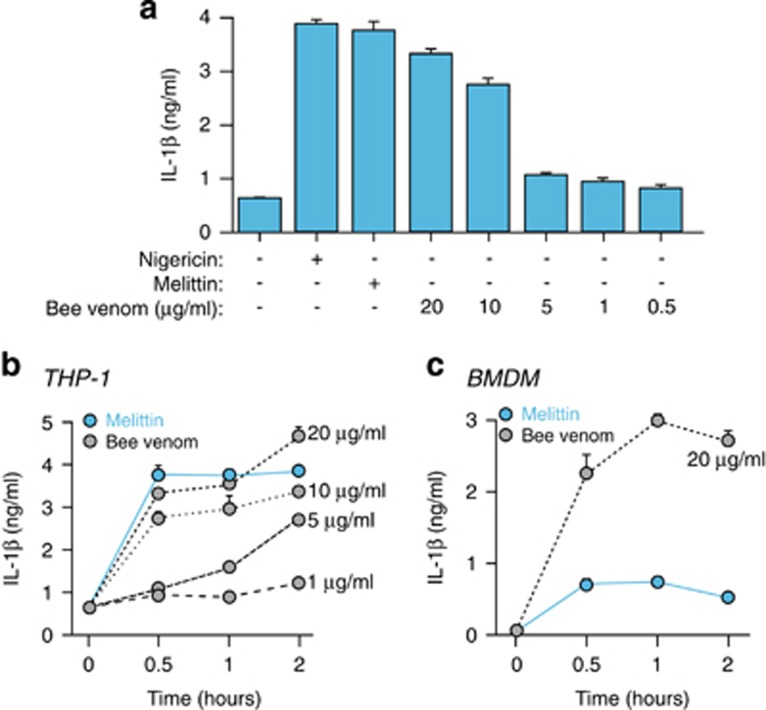
Bee venom induces the release of IL-1*β*. (**a**) ELISA for IL-1*β* release from THP-1 primed with LPS (1 *μ*g/ml, 4 h), and then stimulated or not for 30 min with nigericin (25 *μ*M), melittin (20 *μ*M) or bee venom at the doses shown. (**b** and **c**) Time-course of IL-1*β* release from THP-1 (**b**) or BMDMs (**c**) primed as in panel (**a**), and then stimulated with melittin (20 *μ*M) or bee venom at the doses and for the times shown

Melittin is the main active component of apitoxin (bee venom), and the application of bee venom to lipopolysaccharide (LPS)-primed THP-1 macrophages at concentrations >10 *μ*g/ml induced a robust release of IL-1*β*, comparable to 20 *μ*M of melittin treatment (Figure 2a). Bee venom-induced release of IL-1*β* occurs during the first 30 min of treatment and then reaches a plateau (Figures 2b and c). These kinetics were similar to the kinetics for IL-1*β* melittin-induced release in THP-1 (Figure 2b); however, in mouse BMDMs, bee venom was more potent in inducing IL-1*β* than melittin (Figure 2c), suggesting that other bee venom components could activate different pathways besides melittin in mouse macrophages. Moreover, many antimicrobial peptides have antitumoral activity due to the exposure of phosphatidylserine residues at the surface of these cells, whereas this characteristic is absent in nontransformed cells.^26^ Consequently, susceptibility of THP-1 to other components of the bee venom may differ from BMDMs.

### Melittin activates the NLRP3 inflammasome by potassium efflux

**Figure 3:**
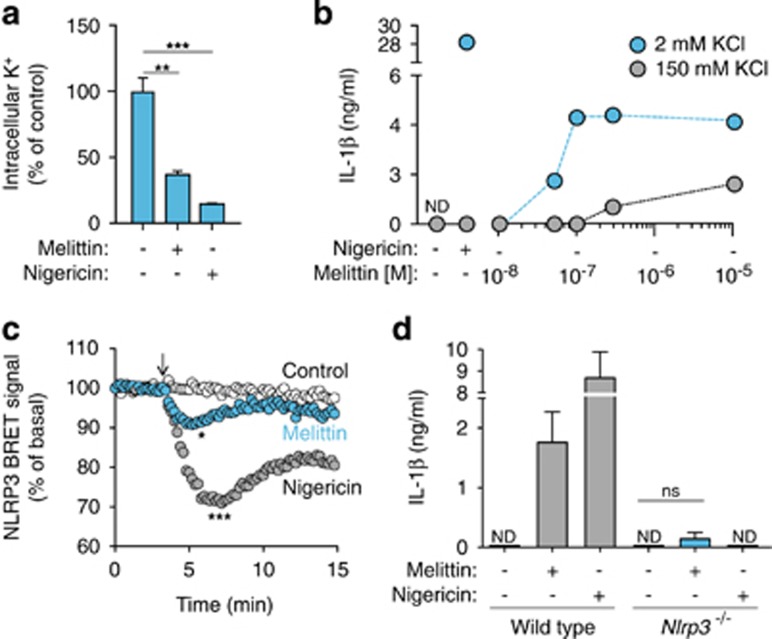
Melittin induces K^+^ depletion and NLRP3 activation. (**a**) Relative intracellular K^+^ concentration from THP-1 primed with LPS (1 *μ*g/ml, 4 h), and then stimulated or not for 30 min with nigericin (25 *μ*M) or melittin (5 *μ*M). (**b**) ELISA for IL-1*β* release from THP-1 primed as in panel (**a**), and then stimulated or not for 30 min with nigericin (25 *μ*M) or melittin in low K^+^ buffer (2 mM KCl, blue circles) or high K^+^ buffer (150 mM KCl, gray circles) at the doses shown. (**c**) Kinetics of net BRET signal for the NLRP3 protein in unstimulated control cells (white circles) or cells treated with nigericin (10 *μ*M, gray circles) or melittin (5 *μ*M, blue circles); nigericin or melittin was added as indicated by the arrow. (**d**) ELISA for IL-1*β* release from wild-type or *Nlrp3*^−/−^ BMDMs primed with LPS (1 *μ*g/ml, 4 h), and then stimulated or not for 30 min with nigericin (25 *μ*M) or melittin (0.5 *μ*M); ND, not detected

**Figure 4:**
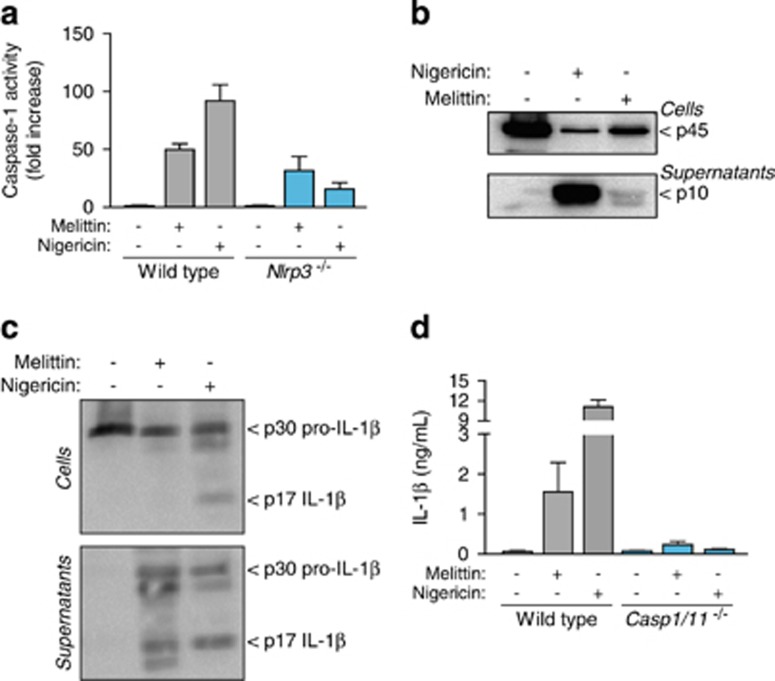
Melittin activates caspase-1 in macrophages. (**a**) Caspase-1 activity in supernatants from wild-type or *Nlrp3*^−/−^ BMDMs primed with LPS (1 *μ*g/ml, 4 h), and then stimulated or not for 30 min with nigericin (25 *μ*M) or melittin (0.5 *μ*M); data are shown as fold increase from unstimulated macrophages for each genotype. (**b** and **c**) Western blot analysis of caspase-1 (**b**) or IL-1*β* (**c**) in cell extracts or supernatants from BMDMs primed with LPS (1 *μ*g/ml, 4 h), and then stimulated or not for 30 min with nigericin (25 *μ*M) or melittin (5 *μ*M). (**d**) ELISA for IL-1*β* release from wild type or *Casp1/11*^−/−^ BMDMs primed and activated as in (**a**)

Low intracellular K^+^ concentration is a key step in NLRP3 activation,^27, 28^ and it has previously reported that bee venom-induced release of IL-1*β* was blocked using a high extracellular K^+^ solution.^5^ In our study, we found that THP-1 macrophage incubation with melittin induced a significant decrease in intracellular K^+^ concentration (Figure 3a). Furthermore, macrophage incubation with melittin in a buffer with a high K^+^ concentration reduced IL-1*β* release from THP-1 macrophages (Figure 3b). An analysis of the NLRP3 bioluminescence resonance energy transfer (BRET) signal during inflammasome activation showed that melittin treatment is able to change the conformation of NLRP3, with a similar but smaller profile than nigericin treatment (Figure 3c). BMDMs from NLRP3-deficient mice failed to release IL-1*β* after melittin treatment (Figure 3d). Melittin also induced an increase in caspase-1 activity in macrophage supernatants that was dependent on NLRP3 (Figure 4a), suggesting that NLRP3 inflammasome was activating caspase-1. The p10 subunit of active caspase-1 was also found in macrophage supernatants after melittin treatment; however, the detection of caspase-1 p10 subunit was weak when compared to nigericin-treated macrophages (Figure 4b). The melittin-induced IL-1*β* that was released was the mature p17 form of the cytokine (Figure 4c) and was dependent on caspase-1 activity (Figure 4d). These data suggest that the decrease in melittin-induced intracellular K^+^ in LPS-primed macrophages triggers the NLRP3 inflammasome to activate caspase-1.

### Melittin does not induce IL-18 release

**Figure 5:**
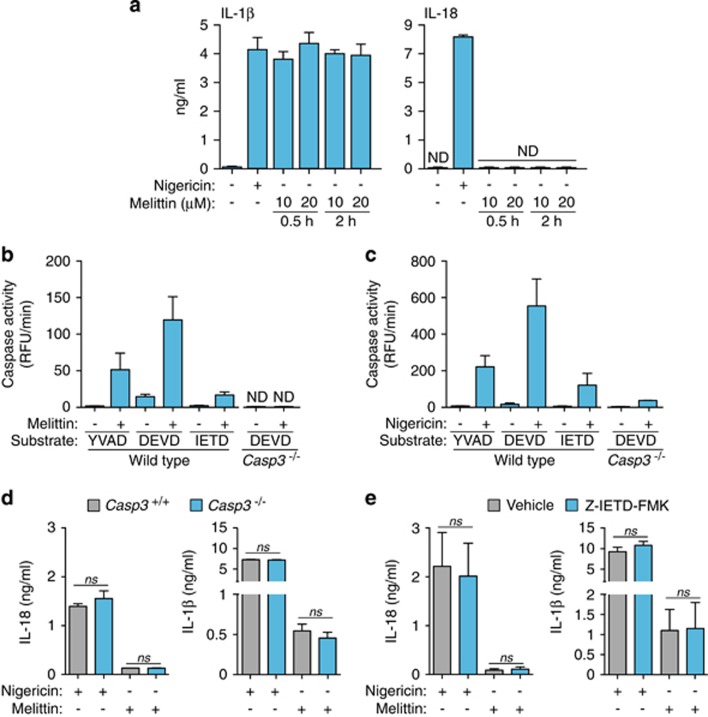
Melittin does not induce IL-18 release. (**a**) ELISA for IL-1*β* or IL-18 release from BMDMs primed with LPS (1 *μ*g/ml, 4 h), and then stimulated or not for 30 min with nigericin (25 *μ*M) or the melittin concentrations and times shown; ND, not detected. (**b** and **c**) Caspase activity measured by the fluorescent peptide substrates indicated in the supernatants from wild type or *Casp3*^−/−^ BMDMs primed with LPS (1 *μ*g/ml, 4 h), and then stimulated or not for 30 min with melittin (0.5 *μ*M) (**b**) or nigericin (25 *μ*M) (**c**). (**d** and **e**) ELISA for IL-1*β* or IL-18 release from wild type (**d**,**e**) or *Casp3*^−/−^ (**d**) BMDMs, treated or not with the caspase-8 inhibitory peptide Z-IETD-FMK (**e**) treated as in (**b** and **c**)

Despite the induction of IL-1*β* release via caspase-1 activation, melittin failed to induce IL-18 release (Figure 5a). The differential secretion of both caspase-1 dependent cytokines was not due to differences in *Il18* gene expression (not shown) and might be explained by specific degradation of IL-18 protein after melittin treatment. It is known that melittin can induce caspase-3 activation,^29^ and caspase-3 can degrade IL-18, resulting in the inactivation of this cytokine.^30^ In macrophages, melittin induced the activation of caspase-3 and to a lesser extent, also of caspase-8 (Figure 5b). However, nigericin also induced the activation of these caspases (Figure 5c) and the release of IL-18 (Figure 5a); hence, it does not seem feasible that caspase-3 can degrade IL-18 in melittin-treated macrophages. To rule out possible degradation of IL-18 by caspase-3, we used caspase-3-deficient macrophages. The deficiency of caspase-3 was not able to rescue IL-18 release from melittin-treated macrophages (Figure 5d). Similarly, caspase-8 inhibition was not able to increase IL-18 release after melittin treatment (Figure 5e). Neither caspase-3 or caspase-8 inhibition affected IL-1*β* or IL-18 release induced by nigericin (Figures 5d and e), denoting a minimal contribution of these caspases to cytokine release after NLRP3 inflammasome activation.

### Melittin does not induce pyroptotic cell death

**Figure 6:**
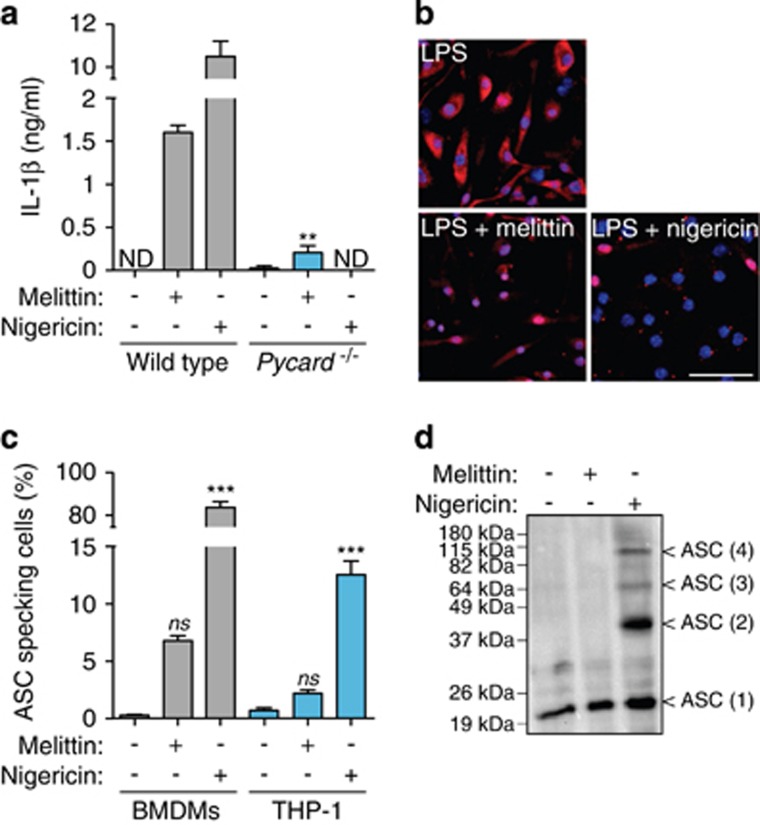
Melittin does not induce ASC oligomerization. (**a**) ELISA for IL-1*β* release from wild type or *Pycard*^−/−^ BMDMs primed with LPS (1 *μ*g/ml, 4 h), and then stimulated or not for 30 min with nigericin (25 *μ*M) or melittin (0.5 *μ*M); ND, not detected. (**b**) Deconvolved representative images of BMDMs treated as in panel (**a**) and stained for ASC (red) and nuclei (blue; DAPI); scale bar, 50 *μ*M. (**c**) Quantification of intracellular ASC speck from BMDMs or THP-1 from pictures as shown in (**b**). (**d**) Western blot from crosslinked ASC from BMDMs stimulated as in (**a**)

The NLRP3 inflammasome requires the adaptor protein ASC to activate caspase-1;^1, 2, 6^ accordingly, melittin-induced IL-1*β* release was reduced in ASC-deficient macrophages (Figure 6a). Canonical NLRP3 inflammasome activation induces ASC oligomerization in large aggregates, which are responsible for amplifying caspase-1 activation and inducing subsequent pyroptotic cell death.^9, 10, 11^ Despite ASC dependence in melittin-induced NLRP3 inflammasome, we only detected less than 10% of melittin-treated macrophages that form ASC specks, compared with the robust and significant induction of ASC specks by nigericin (Figures 6b and c). This was also confirmed by crosslinking experiments, where melittin failed to induce ASC oligomerization (Figure 6d). This suggests that, although melittin may activate caspase-1 via NLRP3 inflammasome using the ASC adaptor protein, ASC failed to form large oligomeric signaling complexes.

**Figure 7:**
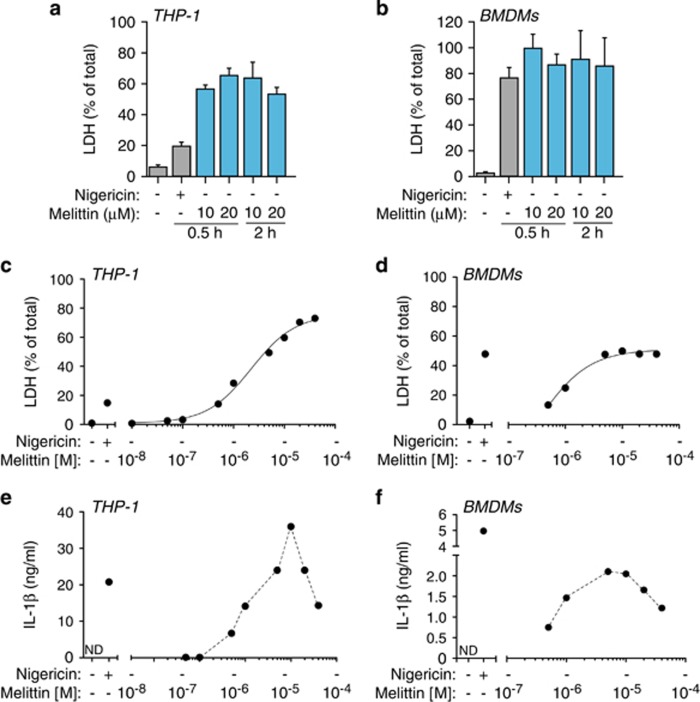
Melittin induces macrophage cell death. (**a** and **b**) Cell death measured as percentage of extracellular LDH in THP-1 (**a**) or BMDMs (**b**) primed with LPS (1 *μ*g/ml, 4 h), and then stimulated or not for 30 min with nigericin (25 *μ*M) or melittin at the concentrations and for the times shown. (**c** and **d**) Dose–response curves for cell death in THP-1 (**c**) or BMDMs (**d**) treated as in (**a** and **b**) for 30 min with the indicated concentrations of melittin. (**e** and **f**) Dose–response for IL-1*β* release from THP-1 (**e**) or BMDMs (**f**) treated as in (**c** and **d**)

**Figure 8:**
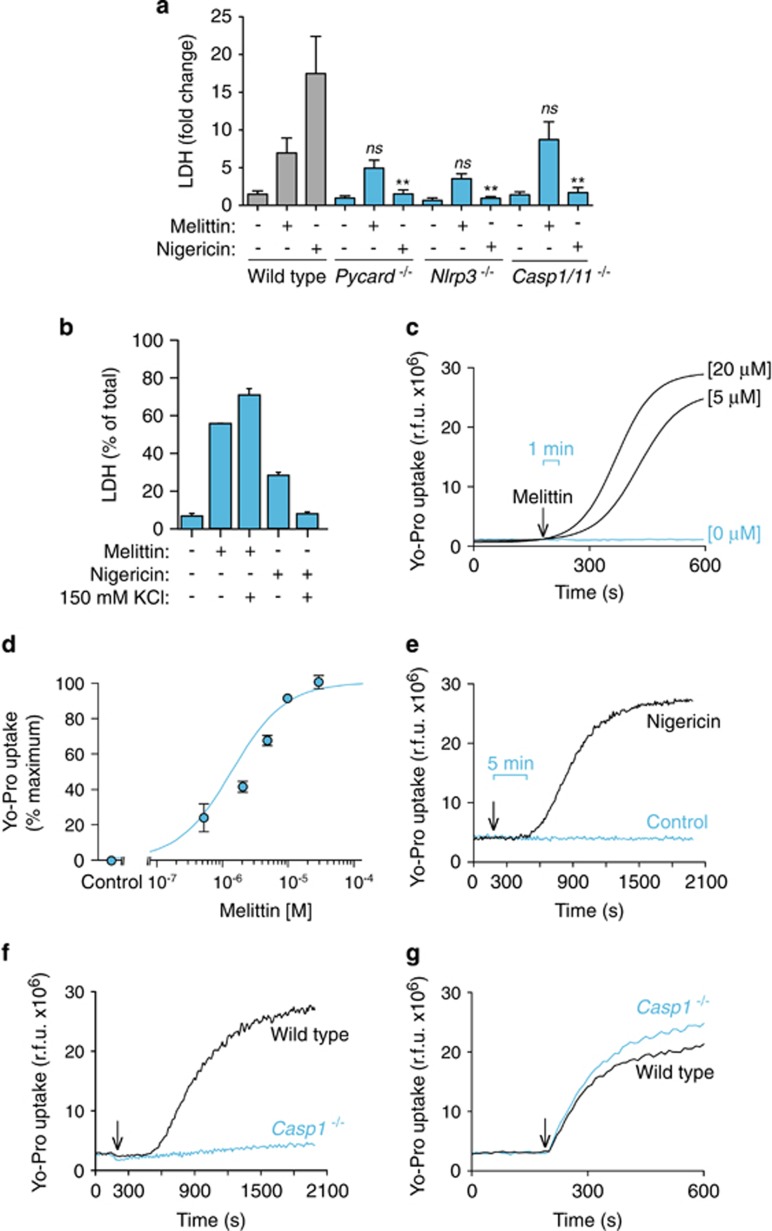
Melittin-induced cell death is independent of the inflammasome. (**a**) Cell death measured as fold increase of extracellular LDH in wild type, *Pycard*^−/−^, *Nlrp3*^−/−^ or *Casp1/11*^−/−^ BMDMs primed with LPS (1 *μ*g/ml, 4 h), and then stimulated or not for 30 min with nigericin (25 *μ*M) or melittin (0.5 *μ*M). (**b**) Cell death measured as fold increase of extracellular LDH in THP-1 primed with LPS (1 *μ*g/ml, 4 h), and then stimulated or not for 30 min with nigericin (25 *μ*M) or melittin (5 *μ*M) in low K^+^ buffer (2 mM KCl) or high K^+^ buffer (150 mM KCl). (**c**) Kinetics of Yo-Pro uptake in BMDMs primed as in (**a**) and stimulated with the melittin concentrations shown; melittin was added as shown by the arrow. (**d**) Dose–response curve for Yo-Pro uptake in BMDMs treated for 20 min as in (**c**) with the melittin concentrations shown. (**e**) Kinetics of Yo-Pro uptake in BMDMs primed as in (**a**) and stimulated with nigericin (5 *μ*M) as shown by the arrow. (**f** and **g**) Kinetics of Yo-Pro uptake in wild type or *Casp1/11*^−/−^ BMDMs primed as in (**a**) and stimulated with nigericin (5 *μ*M) (**f**) or melittin (20 *μ*M) (**g**) as shown by the arrow

The lack of robust ASC oligomerization suggests that melittin might not induce pyroptosis, which is a cell death process dependent on caspase-1. However, melittin induced higher levels of cell death when compared with nigericin treatment (Figures 7a and b). Cell death induced by melittin was dose-dependent reaching a plateau (Figures 7c and d), with a melittin LD_50_ of ∼5 *μ*M in both THP-1 and BMDMs. Similarly, the release of IL-1*β* was dose-dependent for small concentrations of melittin, but cytokine release decreased at high concentrations of melittin (≥ 10 *μ*M) (Figures 7e and f), suggesting that an excessive rate of cell death by rapid cellular lysis leads to reduced NLRP3 inflammasome activation. While nigericin-induced cell death was dependent on NLRP3 and ASC (Figure 8a), melittin-induced cell death was independent on the NLRP3 inflammasome (Figure 8a). In line with this result, caspase-1 deficiency did not affect melittin cell death (Figure 8a). Furthermore, high extracellular K^+^ reduced nigericin but not melittin-induced cell death (Figure 8b). This result confirms that nigericin was inducing pyroptosis dependent on inflammasome and caspase-1 activation, whereas melittin-induced cell death was independent of the inflammasome. Finally, addition of melittin to macrophages immediately induced rapid (≤1 min) plasma membrane permeabilization that was dose-dependent (Figures 8c and d). However, nigericin induced a delayed plasma membrane permeabilization (after 5 min) (Figure 8e), consistent with the time required to activate caspase-1 and induce pyroptosis. In fact, caspase-1 deficiency impaired plasma membrane permeabilization induced by nigericin (Figure 8f), meanwhile melittin-induced plasma membrane permeabilization was independent on caspase-1 (Figure 8g). Altogether, our data show that melittin directly permeabilizes the plasma membrane of macrophages, inducing K^+^ leakage and rapid activation of the NLRP3 inflammasome in order to activate caspase-1 via ASC and induce IL-1*β* maturation and release. Because macrophage viability and plasma membrane integrity were rapidly and directly compromised by melittin, this impairs the subsequent formation of large ASC aggregates and execution of pyroptosis by caspase-1.

## Discussion

We have described that the cytotoxic antimicrobial peptide known as melittin produces plasma membrane disruption with a concomitant decrease in intracellular K^+^ concentration, driving the canonical NLRP3 inflammasome activation. However, melittin-induced plasma membrane permeabilization also leads to rapid cytotoxicity that impairs the formation of large ASC aggregates and the execution of pyroptosis, denoting that necrotic cell death induced by this antimicrobial peptide toxin, similar to other plasma membrane disrupting agents, could preclude pyroptosis.

Antimicrobial peptides are produced by different cells from distinct organisms to aid in the clearance of pathogen infections.^17^ In this study, we examined the function of four cationic natural amphipathic antimicrobial peptides in the activation of the NLRP3 inflammasome: (a) LL-37, the only human cathelicidin peptide produced by a variety of cells, including monocytes; (b) temporin A, an antimicrobial peptide produced by the skin of the European frog *Rana temporaria*; (c) Indolicidin, a peptide present in the cytoplasmic granules of bovine neutrophils; and (d) melittin, a peptide toxin produced by bees and with high microbicidal activity.^17, 23, 31^ The lytic mechanism of these peptides is driven by their small, cationic and amphipathic structures that insert and destabilize membrane structures by different mechanisms.^17^ Although this mechanism of action is shared by these four antimicrobial peptides, we identified melittin as the most active, inducing IL-1*β* release from LPS-primed macrophages, confirming previous work.^5^ LL-37 is also known to induce IL-1*β* release via modulation of the P2X7 receptor in human monocytes;^19^ however, while we also confirm that LL-37 was able to induce IL-1*β* release from human THP-1, it has no activity in mouse macrophages. This may be due to different activation pathways to induce the NLRP3 inflammasome in both cell types; in fact, strong differences were reported in NLRP3 activation after cell swelling when THP-1 and BMDMs were compared.^3^ Furthermore, THP-1 is a derived monocytic cell line, and although treatment with phorbol esters differentiates THP-1 into a macrophage-like type, it is known that monocytes regulate the NLRP3 inflammasome in a different manner.^32^ While in macrophages, NLRP3 requires two-step signaling for its activation, monocytes activate NLRP3 through an alternative pathway that involves the TRIF–RIPK1–FADD–CASP8 pathway upon TLR4 engagement.^33^ Therefore, the differences in activating NLRP3 among monocytes and macrophages could help explain why LL-37 is only able to induce IL-1*β* release in the THP-1 monocytic line. Furthermore, THP-1 is a tumor cell line and, as such, more susceptible to the action of membrane-active antimicrobial peptides than primary cells such as BMDMs.

Melittin is the main component of bee venom and is responsible for inducing an allergic reaction characterized by local inflammation.^21^ NLRP3 inflammasome is known to play a role in this inflammatory response *in vivo*,^5^ and in our study we found that melittin activates the canonical NLRP3 inflammasome pathway via intracellular K^+^ depletion, a common key step for activation of this inflammasome.^27, 28^ NLRP3 activates caspase-1 by recruitment of the adaptor protein ASC via homotypic domain interactions,^6, 7^ and although melittin-induced caspase-1 activation and IL-1*β* secretion required the adaptor ASC protein,^5^ we also found that melittin fails to induce ASC specks, and all cell death driven by melittin was independent of caspase-1. ASC aggregation into large speck-like structures is the result of a prion-like oligomerization process that amplifies inflammasome signaling and executes a specific type of cell death called pyroptosis, driven by gasdermin D cleavage by caspase-1.^9, 10, 11^ The lack of caspase-1-induced pyroptosis after NLRP3 activation by melittin could likely be associated to its rapid effect on plasma membrane destabilization that occurs in parallel to NLRP3 activation. Melittin is a peptide able to induce cell death by the formation of pores in the plasma membrane eukaryotic cells, leading to osmotic cell lysis.^22, 23, 34, 35, 36^ We found plasma membrane destabilization by melittin to occur more rapidly than caspase-1 plasma membrane disruption by the production of the lytic amino-terminal fragment of gasdermin D. However, melittin-induced plasma membrane pore formation is important for inducing caspase-1 activation and IL-1*β* secretion by K^+^ efflux. Similarly, mixed-lineage kinase domain-like protein induces necroptosis and activates the NLRP3 inflammasome, and in this process, IL-1*β* release is independent of gasdermin D plasma membrane permeabilization.^37^ Melittin-induced cell lysis is independent of pyroptosis and excludes pyroptosis because when the plasma membrane is already permeabilized by melittin caspase-1 activates and processes gasdermin D and, therefore, pyroptosis execution by caspase-1 is bypassed. However, melittin is still the initial trigger for NLRP3 activation and IL-1*β* release. It also has been reported that LL-37 inhibits pyroptosis after NLRP3 activation through caspase-1 blocking,^38^ denoting different mechanisms of action for antimicrobial peptides, preventing pyroptosis. Hydroxyapatite crystals also induce the activation of both NLRP3 inflammasome and IL-1*β* release independently of cell lysis, and caspase-1 is not required for hydroxyapatite induced cytotoxicity.^39^

Following caspase-1 activation, together with IL-1*β* there is also a processing and release of the pro-inflammatory cytokine IL-18.^1, 2^ These two inflammasome-derived cytokines, IL-1*β* and IL-18, are differentially induced *in vivo* and may even drive different disease symptoms.^40^ IL-18 has been specifically implicated in the development of age-related macular degeneration, ischemic acute renal failure and NLRC4-associated hyperinflammation.^41, 42, 43^ Furthermore, in mouse models of autoinflammatory cryopyrin-associated periodic syndromes, IL-1*β* and IL-18 drive pathological conditions at different stages of the disease process.^40^ However, *in vitro* both cytokines are usually detected in macrophage supernatants after NLRP3 inflammasome activation, that is, after ATP, nigericin treatment or hypotonicity stimulation, but levels of IL-18 are around 10 times lower than IL-1*β*,^3, 42, 44^ consistent with our findings. Melittin failed to induce IL-18 release, an effect not due to IL-18 degradation by caspase-3, because caspase-3 is a negative regulator mechanism for IL-18, inducing its degradation.^30^ The lack of IL-18 secretion by melittin-treated macrophages could be attributed to low caspase-1 activation induced by melittin treatment, which may be enough to induce IL-1*β*, but not IL-18 release. We surmise that lack of ASC-speck formation, important to amplify caspase-1 activation, may explain this observation.^9^ Overall, melittin induced a differential release of IL-1*β* over IL-18, and this could be important for the resulting response to bee venom. In fact, inflammatory response associated with bee venom has been found to be dependent on IL-1 receptor signaling.^5^

In summary, our work found that the antimicrobial peptide melittin induces the canonical NLRP3 inflammasome activation by permeabilizing the plasma membrane and decreasing intracellular K^+^, but melittin-induced lytic cell death was incompatible with the formation of ASC aggregates, amplification of caspase-1 activation, IL-18 release and execution of pyroptosis. Melittin reveals a pathway for NLRP3 function, where cell death is not driven by caspase-1 and inflammasome signaling is attenuated.

## Materials and methods

### Reagents

The following reagents were used in this study: *Escherichia coli* LPS O55:B5, nigericin and bee venom (Sigma-Aldrich, Madrid, Spain); caspase-8 inhibitor II X-IETD-FMK (Merck-Millipore, Darmstadt, Germany); fluorogenic substrates for caspase-1 YVAD-AFC, caspase-3 DEVD-AFC and caspase-8 IETD-AFC (PromoKine, Heidelberg, Germany); crosslinking reagent SDA (Thermo Scientific, Waltham, MA, USA); rabbit polyclonal antibody against IL-1*β*, caspase-1 p10 (M-20) and anti-ASC (N-15)-R (Santa Cruz Biotechnology, Dallas, TX, USA); ECL horseradish peroxidase-conjugated secondary antibody for immunoblot analysis (GE Healthcare, Uppsala, Sweden); and Alexa Fluor 647-conjugated donkey anti rabbit IgG secondary antibody and ProLong Diamond Antifade Mountant with 4′,6-diamidino-2-phenylindole (DAPI) (Life Technologies, Paisley, UK).

### Cell culture

Bone marrow was obtained from wild-type C57BL/6, *Nlrp3*^−/−^, *Pycard*^−/−^ or *Casp1*^−/−^*Casp11*^−/−^ mice^4, 45, 46^ and differentiated to bone marrow derived-macrophages (BMDMs) using standard protocols.^47^ All animals were kept under controlled pathogen-free conditions (20±2 °C and 12-h light–dark cycle), with free access to sterile food and water. THP-1 cells were stored in Roswell Park Memorial Institute (RPMI) 1640 media and human embryonic kidney 293 (HEK293) cells in Dulbecco’s modified Eagle’s medium media, both supplemented with 10% fetal calf serum). Cell lines were routinely checked to ensure they were mycoplasma-free.

### Peptides

The antimicrobial peptides tested (Table 1) were produced by solid-phase synthesis in ABI433 (Applied Biosystems, Foster City, CA, USA) or Prelude (Protein Technologies, Manchester, UK) synthesizers running optimized Fmoc protocols. After assembly, the peptide resins were treated with TFA/H_2_O/triisopropylsilane (95:2.5:2.5, by vol.) for 90 min for side-chain deprotection and cleavage from the resin. The crude peptides were isolated by precipitation with cold ether and centrifugation (3000 × *g*, 20 min, 4 °C), dissolved in 0.1 M acetic acid, lyophilized and purified by reverse-phase HPLC to >95% homogeneity. The identity of these peptides was confirmed by matrix-assisted laser desorption/ionization coupled time-of-flight mass spectrometry (MALDI–TOF MS) using *α*-hydroxycinnamic acid as the matrix.^18^

### Enzyme-linked immunosorbent assay

IL-1*β* and IL-18 release were measured using the respective kits for human or mouse (R&D Systems, Minneapolis, MN, USA) following the manufacturer’s instructions.

### Lactate dehydrogenase-release measurements

Pyroptotic cell death was determined by measuring the amount of extracellular lactate dehydrogenase (LDH) using the Cytotoxicity Detection Kit (Roche, Mannheim, Germany) following the manufacturer’s instructions. Extracellular LDH activity was expressed as a percentage of the total amount of intracellular LDH under nonstimulated conditions.

### Caspase activity

Supernatants from stimulated cells were monitored every 30 min for 6 h for the cleavage of fluorescent substrates for caspase-1 YVAD-AFC, caspase-3 DEVD-AFC or caspase-8 IETD-AFC, at 400 nm excitation and 505 nm emission using a Synergy Mx plate reader (BioTek, Winooski, VT, USA).

### Yo-Pro uptake

Cells were incubated with 2.5 *μ*M Yo-Pro-1 (Life Technologies) for 5 min, and fluorescence was recorded every 10 s for 35 min at 37 ºC, before and after the addition of melittin or nigericin at different concentrations as annotated in the figure legends. Fluorescence of DNA-bound Yo-Pro-1 was measured in a Synergy Mx plate reader (BioTek) at 485±9 nm excitation and 515±9 nm emission.

### Intracellular K^+^ determination

THP-1 cells were lysed in ultrapure water by five freezing–thaw cycles. The lysates were centrifuged, and K^+^ was quantified by indirect potentiometry using a Cobas 6000 with ISE module (Roche).

### ASC crosslinking and western blot

ASC crosslinking was performed following the standard procedure.^48^ Briefly, cells were lysed with (3-((3-cholamidopropyl) dimethylammonio)-1-propanesulfonate) (CHAPS) buffer (20 mM (4-(2-hydroxyethyl)piperazine-1-ethanesulfonic acid potassium salt (HEPES-KOH) pH 7.5, 5 mM MgCl_2_, 0.5 mM ethylene glycol-bis(β-aminoethyl ether)-*N*,*N*,*N′*′,N′-tetraacetic acid, 0.1% CHAPS and incubated 45 min with 2 mM succinimidyl 4,4′-azipentanoate (SDA), then quenched with 50 mM Tris-HCl pH 8.8 for 15 min and crosslinked for 15 min under 365 nm UV light. Crosslinked samples, or cell lysates and concentrated cell-free supernatants were all resolved in 4–12% polyacrylamide gels and electrotransferred. Membranes were probed with different antibodies for ASC, IL-1*β* or caspase-1.

### BRET assay

HEK293 cells were transfected with a vector encoding for human NLRP3 tagged with luciferase (N-terminus) and YFP (C-terminus)^49^ using Lipofectamine 2000 (Invitrogen, Carlsbad, CA, USA) following the manufacturer’s instructions. After 24 h, transfected cells were seeded on a poly-l-lysine-coated white 96-well plate the day before the assay. The BRET signal was read after 5 min of coelenterazine-h (5 *μ*M; Invitrogen) addition. Luminescence was detected at 37 °C in a Synergy Mx plate reader (Biotek) using two filters for 485±20  and 528±20 nm emission. The BRET ratio was calculated as the difference between the 528 and 485 nm emission ratio of R-Luc and YFP-NLRP3 fusion protein and the 530 and 485 nm emission ratio of the R-Luc protein alone. Results are expressed in milliBRET (mBRET) units normalized to the basal signal, as we have previously described.^50^

### Immunofluorescence

Macrophages were seeded on poly-l-lysine coverslips 24 h before use. After stimulation, cells were fixed with 4% formaldehyde, blocked using 1% bovine serum albumin (Sigma-Aldrich) and permeabilized with 0.2% saponin (Sigma-Aldrich). Cells were stained using the rabbit polyclonal antibody anti-ASC (1:500 dilution) and the Alexa Fluor 467 conjugated secondary antibody (1:200 dilution). The coverslips were mounted on slides with ProLong Diamond Antifade Mountant with DAPI. Images were acquired with an Eclipse Ti microscope (Nikon, Tokyo, Japan) equipped with a × 20 S Plan Fluor objective (numerical aperture, 0.45), a × 40 S Plan Fluor objective (numerical aperture, 0.6) and a × 60 S Plan Apo Vc objective (numerical aperture, 1.40) and a digital Sight DS-QiMc camera (Nikon) with a Z optical spacing of 0.2 *μ*M and 387 /447, 482/536, 543/593 and 650 nm/668 nm filter sets (Semrock, Lake Forest, IL, USA). Images were processed using ImageJ software (NIH-National Institutes of Health (Bethesda, MD, USA)).

### Statistical analysis

Data are shown as the mean±S.E.M. from at least three independent experiments. The data were analyzed using Prism (GraphPad, La Jolla, CA, USA) software, by one-way ANOVA with Bonferroni multiple-comparison post-test to determine the differences among more than two groups (***P<0.001; **P<0.01; *P<0.05; NS, not significant (*P*>0.05) difference).

## Figures and Tables

**Table 1:** Peptide sequences used in this study

**Name**	**Sequence (amino-terminal>carboxyl-terminal)**
Temporin A	FLPLIGRVLSGIL
LL-37	LLGDFFRKSKEKIGKEFKRIVQRIKDFLRNLVPRTES
Indolicidin	ILPWKWPWWPWRR
Cecropin A	KWKLFKKIEKVGQNIRDGIIKAGPAVAVVGQATQIAK
Melittin	GIGAVLKVLTTGLPALISWIKRKRQQ
(1) CA(1–8)M(1–18)	KWKLFKKIGIGAVLKVLTTGLPALIS
(2) CA(1–7)M(2–9)	KWKLFKKIGAVLKVL
(3) CA(1–7)M(5–9)	KWKLFKKVLKVL
(4) [^16^d-Val, ^17^d-Leu] CA(1–8)M(1–18)	KWKLFKKIGIGAVLK**vl**TTGLPALIS
(5) [^4^D-Leu, ^5^D-Phe] CA(1–8)M(1–18)	KWK**lf**KKIGIGAVLKVLTTGLPALIS

All peptides except LL-37 are C-terminal carboxamides. In cecropin A (CA)-melittin (M) chimeric peptides, the underlined regions correspond to the melittin sequence. Lowercase and bold type text in the diastereomeric peptide sequences (4) and (5) indicates d-amino acid residues.
